# Prevalence, risk factors, and cardiovascular disease outcomes associated with persistent blood pressure control: The Jackson Heart Study

**DOI:** 10.1371/journal.pone.0270675

**Published:** 2022-08-05

**Authors:** Gabriel S. Tajeu, Calvin L. Colvin, Shakia T. Hardy, Adam P. Bress, Bamba Gaye, Byron C. Jaeger, Gbenga Ogedegbe, Swati Sakhuja, Mario Sims, Daichi Shimbo, Emily C. O’Brien, Tanya M. Spruill, Paul Muntner

**Affiliations:** 1 Department of Health Services Administration and Policy, Temple University, Philadelphia, PA, United States of America; 2 Department of Epidemiology, University of Alabama at Birmingham, Birmingham, AL, United States of America; 3 Department of Population Health Sciences, University of Utah School of Medicine, Salt Lake City, UT, United States of America; 4 INSERM, U970, Paris Cardiovascular Research Center, Department of Epidemiology, Paris, France; 5 Sorbonne Paris Cité, Faculté de Médecine, Université Paris Descartes, Paris, France; 6 Department of Biostatistics and Data Science, Wake Forest University School of Medicine, Wake Forest, NC, United States of America; 7 Department of Population Health, NYU Grossman School of Medicine, New York, NY, United States of America; 8 Department of Medicine, University of Mississippi Medical Center, Jackson, MS, United States of America; 9 Department of Medicine, Columbia University Irving Medical Center, New York, NY, United States of America; 10 Departments of Population Health Sciences and Neurology, Duke University School of Medicine, Durham, NC, United States of America; Albert Einstein College of Medicine, UNITED STATES

## Abstract

**Background:**

Maintaining blood pressure (BP) control over time may contribute to lower risk for cardiovascular disease (CVD) among individuals who are taking antihypertensive medication.

**Methods:**

The Jackson Heart Study (JHS) enrolled 5,306 African-American adults ≥21 years of age and was used to determine the proportion of African Americans that maintain persistent BP control, identify factors associated with persistent BP control, and determine the association of persistent BP control with CVD events. This analysis included 1,604 participants who were taking antihypertensive medication at Visit 1 and had BP data at Visits 1 (2000–2004), 2 (2005–2008), and 3 (2009–2013). Persistent BP control was defined as systolic BP <140 mm Hg and diastolic BP <90 mm Hg at all three visits. CVD events were assessed from Visit 3 through December 31, 2016. Hazard ratios (HR) for the association of persistent BP control with CVD outcomes were adjusted for age, sex, systolic BP, smoking, diabetes, and total and high-density lipoprotein cholesterol at Visit 3.

**Results:**

At Visit 1, 1,226 of 1,604 participants (76.4%) with hypertension had controlled BP. Overall, 48.9% of participants taking antihypertensive medication at Visit 1 had persistent BP control. After multivariable adjustment for demographic, socioeconomic, clinical, behavioral, and psychosocial factors, and access-to-care, participants were more likely to have persistent BP control if they were <65 years of age, women, had family income ≥$25,000 at each visit, and visited a health professional in the year prior to each visit. The multivariable adjusted HR (95% confidence interval) comparing participants with versus without persistent BP control was 0.71 (0.46–1.10) for CVD, 0.68 (0.34–1.34) for coronary heart disease, 0.65 (0.27–1.52) for stroke, and 0.55 (0.33–0.90) for heart failure.

**Conclusion:**

Less than half of JHS participants taking antihypertensive medication had persistent BP control, putting them at increased risk for heart failure.

## Introduction

Hypertension is a major modifiable risk factor for cardiovascular disease (CVD [1–3]. Antihypertensive medication has been shown to lower blood pressure (BP) and reduce the risk for CVD among adults with hypertension [4]. Among individuals taking antihypertensive medication, those with controlled BP have a lower risk for CVD events compared to their counterparts with uncontrolled BP [5, 6]. However, according to the US National Health and Nutrition Examination Survey (NHANES), only 65% of US adults taking antihypertensive medication had systolic BP (SBP) <140 mm Hg and diastolic BP (DBP) <90 mm Hg in 2017–2018 [7].

NHANES only provides cross-sectional estimates of BP control at a single time point [8, 9]. However, prior studies report that many people with BP control at a single time point do not have BP control when assessed at multiple visits [10], what we refer to in the current manuscript as persistent BP control. In a secondary analysis of the Antihypertensive Lipid-Lowering to prevent Heart Attack Trial (ALLHAT), only 20% of participants had controlled SBP at eight follow-up visits conducted over a 22 month period [10]. ALLHAT participants were treated with antihypertensive medication following a standardized protocol and few data on persistent BP control over time are available from population-based studies. Therefore, it is unclear what percentage of people with hypertension in the general population versus those in a clinical trial maintain persistent BP control over time.

Among US adults with hypertension, African Americans are less likely than whites to have controlled BP and have a higher risk for hypertension-related CVD including stroke and heart failure [6]. Studying persistent BP control among African-American adults has the potential to inform interventions to improve BP control and reduce racial disparities in CVD. Therefore, the purpose of this study was to determine the percentage of African-American adults with persistent BP control, identify factors associated with persistent BP control, and determine the association of persistent BP control with risk for CVD outcomes. To address these goals, we analyzed data from the Jackson Heart Study (JHS), a community-based cohort of African-American adults.

## Methods

### Study population

The JHS was designed to determine the reasons for the high prevalence of CVD in African-Americans and identify approaches for reducing this risk [11]. Between 2000 and 2004, 5,306 non-institutionalized African-American adults ≥21 years of age were enrolled into the JHS. Participants were recruited from the three counties (Hinds, Madison, and Rankin) that comprise the Jackson, Mississippi metropolitan area. To date, there have been three JHS visits including baseline (Visit 1) from 2000–2004, Visit 2 from 2005–2008, and Visit 3 from 2009–2013. We restricted the analysis of the proportion of participants with persistent BP control and factors associated with persistent BP control to those who were taking antihypertensive medication at Visit 1 and had complete data on SBP and DBP at Visit 1, Visit 2, and Visit 3 (**S1 Fig in S1 File**). Antihypertensive medication use was defined by self-report and confirmed by a review of prescription pill bottles that was conducted by study staff. After applying these criteria, 1,604 participants were included in the analysis. For the analysis examining the association of persistent BP control with CVD events following Visit 3, we further excluded participants with a history of CVD at Visit 1, those who did not consent to follow-up for CVD events and those who had a CVD outcome between Visits 1 and 3. Overall, 1,151 participants were included in the analysis of persistent BP control and CVD events. The JHS was approved by the institutional review boards of the University of Mississippi Medical Center, Jackson State University, and Tougaloo College, and all participants provided written informed consent at each visit.

### Persistent BP control

At each study visit, BP was measured by trained staff following a standardized protocol while participants were seated and after a five-minute rest. Participants’ right arms were fitted with an appropriately-sized cuff and two BP measurements were taken, with a one minute interval separating the measurements. Staff used a random-zero sphygmomanometer (Hawksley and Sons, Ltd, London, UK) to measure BP at Visits 1 and 2, and a semi-automated oscillometric device (Omron HEM-907XL, Omron Healthcare Inc., Lake Forest, IL) at Visit 3. BP measurements performed using the random-zero sphygmomanometer were calibrated to the oscillometric device after the completion of a BP comparability study [12]. The mean BP from each visit was used in the analyses. At each visit, controlled BP was defined as SBP <140 mm Hg and DBP <90 mm Hg. Persistent BP control was defined as having controlled BP at all three study visits.

### Baseline factors

Demographic factors included age, sex, education level and marital status. The number of antihypertensive medication classes taken at baseline was included as a clinical factor. Cigarette smoking at baseline was included as a behavioral factor, and we included health insurance status at baseline as a measure of access to healthcare. We included weekly stress and depressive symptoms at baseline as psychosocial factors. **S1 Table in S1 File** lists the definitions of and the methods used to assess baseline factors.

### Time-varying factors

Several factors were available at multiple JHS visits (**S1 Table in S1 File**). Annual family income was included as a socioeconomic factor. Body mass index (BMI) and measures of glycemic control were included as clinical factors. Behavioral factors included adherence to antihypertensive medication, alcohol consumption, and physical activity. Participant access to healthcare included annual healthcare visits and difficulty accessing healthcare. Anger expression and daily discrimination were included as psychosocial factors. For each study visit, we categorized participants as having ideal or non-ideal levels of each of these factors (see **S1 Table in S1 File**). We then categorized participants as maintaining ideal levels of each factor if they were in the ideal category at all visits at which they were assessed.

### Incident CVD events

The primary CVD outcome was a composite of coronary heart disease (CHD: i.e., myocardial infarction, fatal CHD, or a cardiac procedure), stroke, and heart failure. CHD, stroke and heart failure were investigated individually as secondary CVD outcomes. CVD events were identified by annual telephone follow-up interviews, hospitalization surveillance, and death certificate review. Possible events were then adjudicated by trained abstractors. A detailed description of the JHS follow-up and CVD event adjudication process has been published previously [13].

### Statistical analysis

We estimated summary statistics for participants with and without persistent BP control, separately. We calculated the proportion of participants with persistent BP control, overall and by levels of baseline and time-varying factors. We used two Poisson regression models with robust variance estimates to calculate risk ratios (RR) and 95% confidence intervals (CI) for the association of baseline and time-varying factors with persistent BP control. Model 1 included adjustment for age and sex and each baseline and time-varying factor listed above one at a time. Model 2 included all baseline and time-varying factors. Multiple imputation with chained equations was applied to impute missing values (**S2 Table in S1 File**). Imputation models were regression based and used all analysis variables to impute missing data. A total of 10 imputed datasets were created and results were pooled to obtain valid standard error estimates. All calculations were done for the overall included population and for those with controlled BP at baseline.

We calculated incidence rates and hazard ratios (HR) with 95% CIs for CVD, CHD, stroke, and heart failure among participants with and without persistent BP control. For the calculation of incidence rates and HRs, participants were followed from the date of their Visit 3 examination through the date of their first CVD event with censoring occurring for those who remained event free on the date of their last contact with the JHS, date of death, or December 31, 2016, whichever occurred first. Two models with progressive adjustment were used to calculate HRs. Model 1 included adjustment for age and sex. Model 2 included adjustment for age, sex, SBP, smoking, diabetes, total cholesterol, and high-density lipoprotein (HDL) cholesterol measured at Visit 3. These variables are included in the Pooled Cohort Risk Equations which is used to assess 10-year CVD risk [14] and we adjusted for these variables due to their robust association with CVD event risk [14]. In a secondary analysis, we calculated the incidence rates and HRs for CVD, CHD, stroke, and heart failure comparing participants with persistent BP control across all three study visits to participants with controlled BP at Visit 3 but without persistent BP control. Statistical analyses were conducted using SAS Version 9.4 (SAS Institute, Cary, NC) and STATA Version 16.1 (StataCorp, College Station, TX).

## Results

At Visit 1, 76.4% (n = 1,226 of 1,604) of participants included in the current analysis had controlled BP. Among those with controlled BP at Visit 1, 64.0% had persistent BP control (i.e., controlled BP at all three visits). Overall, 785 of 1,604 (48.9%) participants had persistent BP control over a median follow up time of 8.0 years (25^th^-75^th^ percentile: 7.4–8.3 years). Characteristics of participants with and without persistent BP control are presented in **Table 1**.

**Table 1:** Baseline characteristics of Jackson Heart Study participants taking antihypertensive medication by persistent blood pressure control status.

Characteristics	Persistent blood pressure control	
	Yes n = 785	No n = 819
Demographic		
Age in years, mean (SD)	57.1 (9.8)	60.6 (10.2)
Men, %	28.8	31.9
Income <$25,000 per year, %	32.4	41.3
Less than high school education, %	14.7	22.1
Married, %	57.7	54.9
Clinical factors		
Systolic blood pressure, mean (SD)	122 (10)	138 (16)
Diastolic blood pressure, mean (SD)	74.1 (7.6)	77.9 (9.4)
Number of antihypertensive medication classes, %		
1	27.0	30.2
2	45.2	40.2
3	19.4	19.8
≥4	8.4	9.9
Body mass index < 25 kg/m^2^, %	6.9	8.7
Ideal glycemic control, %	31.3	30.9
Behavioral factors		
Adherence to antihypertensive medication, %	78.7	71.2
Cigarette smoking, no, %	91.6	91.9
Alcohol consumption, no, %	59.0	62.3
Ideal physical activity, %	20.3	17.3
Access to health care		
Health insurance, %	91.1	89.0
Healthcare visit in the past year, %	86.1	84.8
Difficulty in obtaining health services, %	27.1	27.2
Psychosocial factors		
Stress, %		
Low	35.1	31.9
Moderate	31.9	34.6
High	33.0	33.5
Depressive symptoms, %	19.0	23.4
Anger-in, %		
Low	37.0	34.6
Moderate	36.2	34.4
High	26.8	31.0
Anger-out, %		
Low	32.2	30.4
Moderate	40.7	37.9
High	27.1	31.8
Daily discrimination, %		
Quartile 1 (low)	26.0	27.5
Quartile 2	21.0	21.7
Quartile 3	28.1	25.8
Quartile 4 (high)	24.9	25.0

SD = standard deviation.

Stress scale tertile cut points: low (0–31), moderate (32–80), high (81–482)

Anger-in scale tertile cut points: low (8–11), moderate (12–14), high (15–28)

Anger-out scale tertile cut points: low (8–10), moderate (11–13), high (14–29)

Daily discrimination scale quartile cut points: quartile 1 (1.00–1.32), quartile 2 (1.33–1.76), quartile 3 (1.77–2.54), quartile 4 (2.55–7.00).

### Prevalence of persistent BP control by participant characteristics

Participants who were <65 years of age, maintained an income ≥$25,000, had a high school education, were adherent to their antihypertensive medication across the three study visits, drank alcohol, and had visited a healthcare professional in the year before each study visit were more likely to have persistent BP control (**Table 2**). Each of these factors, except being adherent to antihypertensive medication and drinking alcohol, was associated with persistent BP control among participants with controlled BP at baseline (**S3 Table in S1 File**).

**Table 2:** Percentage of participants with persistent blood pressure control in sub-groups.

Characteristics	Percentage with persistent BP control	p-value
Overall	48.9	
Demographic		
Age		
<65 years	53.4	<0.001
≥65 years	38.8	
Sex		
Men	46.4	0.19
Women	50.0	
Maintained income ≥$25,000 per year*		
No	43.8	<0.001
Yes	56.1	
High school education		
No	38.9	<0.001
Yes	51.2	
Marital status		
Married	50.2	0.29
Not married	47.4	
Clinical factors		
Number of antihypertensive medication classes		
1	46.2	0.19
2	51.9	
3	48.4	
≥4	44.9	
Maintained ideal body mass index*		
No	49.9	0.47
Yes	45.2	
Maintained ideal glycemic control*		
No	49.7	0.69
Yes	51.9	
Behavioral factors		
Maintained adherence to antihypertensive medication*		
No	46.6	0.02
Yes	52.7	
Cigarette smoking		
No	48.9	0.86
Yes	50.0	
Maintained ideal alcohol consumption status*		
No	51.9	0.03
Yes	46.5	
Maintained ideal physical activity*		
No	49.3	0.93
Yes	48.8	
Access to health care		
Health insurance		
Uninsured	43.8	0.18
Insured	49.5	
Reported visiting a healthcare professional in the past year at each study visit*		
No	43.6	0.003
Yes	51.8	
Maintained no difficulty in obtaining health services*		
No	47.8	0.21
Yes	51.0	
Psychosocial factors		
Stress		
Low	53.5	0.54
Moderate	49.0	
High	50.7	
Depression		
No depressive symptoms	51.1	0.09
Depressive symptoms	44.6	
Maintained ideal anger-in*		
No	49.6	0.86
Yes	50.7	
Maintained ideal anger-out*		
No	50.3	0.53
Yes	47.3	
Maintained low levels of daily discrimination*		
No	49.9	0.07
Yes	43.0	

BP = blood pressure

*These factors were available at multiple study visits. For each study visit where these variables were available, we categorized participants as having ideal or non-ideal levels of each of these factors. We then categorized participants as maintaining ideal levels of each factor if participants were in the ideal category at all visits in which they were collected. **S1 Table in S1 File** lists these study variable definitions, visits at which they were collected, collection methods, and their classification for ideal level status.

Stress scale tertile cut points: low (0–31), moderate (32–80), high (81–482)

### Adjusted associations of participant characteristics with persistent BP control

After adjustment for sex, participants ≥65 years of age were less likely to have persistent BP control compared with participants <65 years of age (**Table 3 –Left Panel**). After age and sex adjustment, participants were more likely to have persistent BP control if they maintained a family income ≥ $25,000 a year, had a high school education, maintained adherence to antihypertensive medication, had health insurance at baseline, and visited a health professional in the year prior to each study visit. In a model with all demographic, clinical, behavioral, access to healthcare and psychosocial factors, participants ≥65 years of age were less likely to have persistent BP control, while women, participants who maintained an income ≥ $25,000 a year, and who reported visiting a health professional in the year before each study visit were more likely to have persistent BP control (**Table 3 –Right Panel**). **S4 Table in S1 File** presents the RRs for persistent BP control associated with participant characteristics among JHS participants with controlled BP at Visit 1.

**Table 3:** Adjusted risk ratios for persistent blood pressure control among participants taking antihypertensive medication (n = 1,604).

Characteristic	Risk ratio (95% CI) Model 1	p-value	Risk ratio (95% CI) Model 2	p-value
Demographic				
Age: ≥65 years compared to <65 years	0.73 (0.64–0.82)	<0.001	0.77 (0.67–0.88)	<0.001
Sex: women vs men	1.08 (0.97–1.21)	0.155	1.13 (1.01–1.28)	0.035
Maintained income ≥$25,000 per year*: yes vs no	1.28 (1.14–1.43)	<0.001	1.19 (1.04–1.35)	0.012
High school education: yes vs. no	1.20 (1.03–1.41)	0.020	1.06 (0.90–1.26)	0.494
Marital status: married vs not married	1.08 (0.97–1.19)	0.160	1.01 (0.91–1.12)	0.867
Clinical factors				
Number of antihypertensive medication classes				
1	Ref		Ref	
2	1.12 (0.99–1.26)	0.073	1.13 (1.00–1.27)	0.053
3	1.05 (0.90–1.22)	0.533	1.05 (0.91–1.22)	0.490
≥4	0.96 (0.78–1.18)	0.708	0.97 (0.79–1.19)	0.797
Maintained ideal body mass index*: yes vs no	0.94 (0.73–1.22)	0.663	0.95 (0.74–1.24)	0.727
Maintained ideal glycemic control*: yes vs no	0.96 (0.82–1.14)	0.658	0.94 (0.79–1.11)	0.456
Behavioral factors				
Maintained adherence to antihypertensive medication: yes vs. no*	1.11 (1.01–1.23)	0.037	1.09 (0.99–1.20)	0.094
Current cigarette smoking: yes vs. no	0.97 (0.81–1.16)	0.752	1.02 (0.85–1.23)	0.824
Maintained non-drinker status*: yes vs. no	0.93 (0.84–1.03)	0.184	0.97 (0.87–1.08)	0.554
Maintained ideal physical activity*: yes vs. no	0.99 (0.82–1.20)	0.929	0.94 (0.78–1.14)	0.537
Access to health care				
Health insurance: insured vs. uninsured	1.24 (1.03–1.49)	0.025	1.12 (0.92–1.36)	0.256
Reported visiting a healthcare professional in the past year at each study visit*: yes vs no	1.23 (1.09–1.37)	<0.001	1.19 (1.06–1.34)	0.003
Maintained no difficulty in obtaining health services*: yes vs no	1.08 (0.98–1.20)	0.112	1.00 (0.90–1.10)	0.933
Psychosocial factors				
Stress				
Low	Ref		Ref	
Moderate	0.93 (0.80–1.08)	0.336	0.95 (0.81–1.10)	0.481
High	0.95 (0.81–1.12)	0.557	1.01 (0.86–1.20)	0.858
Depressive symptoms vs with no symptoms	0.86 (0.73–1.01)	0.063	0.91 (0.76–1.08)	0.266
Maintained ideal anger-in*: yes vs no	1.04 (0.89–1.21)	0.646	1.01 (0.86–1.18)	0.945
Maintained ideal anger-out*: yes vs no	0.99 (0.84–1.17)	0.941	1.01 (0.85–1.19)	0.915
Maintained low levels of daily discrimination*: yes vs no	0.94 (0.80–1.11)	0.448	0.95 (0.80–1.12)	0.522

CI = confidence interval

Model 1 adjusted for age and sex.

Model 2 adjusted for all variables listed in the table.

*These factors were available at multiple study visits. For each study visit where these variables were available, we categorized participants as having ideal or non-ideal levels of each of these factors. We then categorized participants as maintaining ideal levels of each factor if participants were in the ideal category at all visits in which they were collected. **S1 Table in S1 File** lists these study variable definitions, visits at which they were collected, collection methods, and their classification for ideal level status.

Stress scale tertile cut points: low (0–31), moderate (32–80), high (81–482)

### Persistent BP control and risk for incident CVD

**Fig 1:**
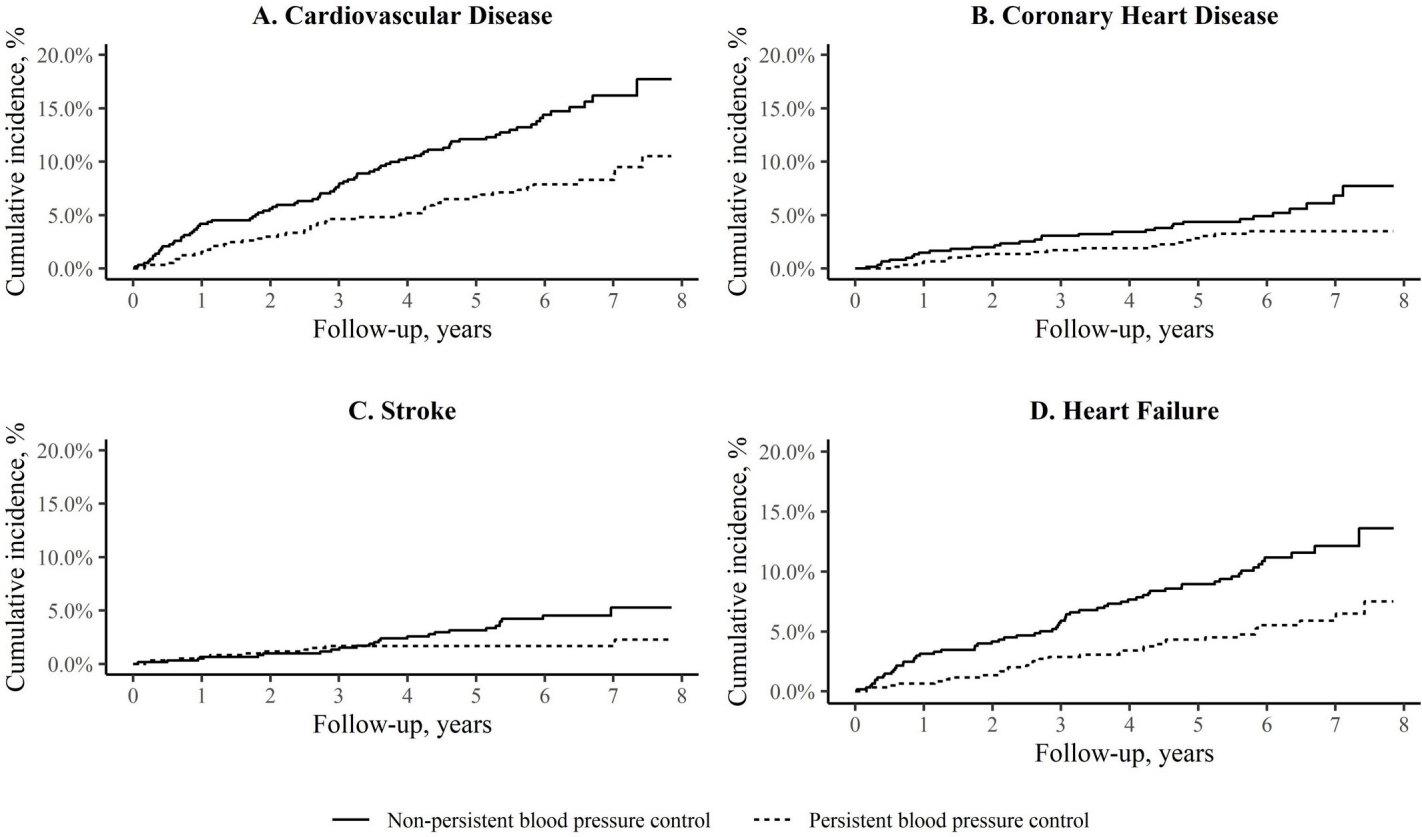
Cumulative incidence of cardiovascular disease events among adults taking antihypertensive medication. Persistent blood pressure (BP) control was defined as having controlled BP (systolic BP <140 mm Hg and diastolic BP <90 mm Hg) across three Jackson Heart Study visits (Visit 1 from 2000–2004, Visit 2 from 2005–2008, and Visit 3 from 2009–2013). Cardiovascular disease was a composite of coronary heart disease, stroke, and heart failure.

Among 1,151 participants without CVD at Visit 3, there were 127 incident CVD events over a median follow-up time of 6.0 years (25^th^-75^th^ percentile: 5.2–6.9). The incidence rate for CVD was 13.7 (95% CI: 9.8–17.7) and 25.6 (95% CI: 20.1–31.2) per 1,000 person-years among participants with and without persistent BP control (**Table 4**). The cumulative incidence for CVD, CHD, stroke, and heart failure by persistent BP control status are presented in the **Fig 1**. After multivariable adjustment for age, sex, diabetes, current smoking, SBP, total cholesterol and HDL cholesterol, the HR for CVD comparing participants with versus without persistent BP control was 0.71 (95% CI: 0.46–1.10) (**Table 4**). The multivariable adjusted HRs for CHD, stroke, and heart failure were 0.68 (95% CI: 0.34–1.34), 0.65 (95% CI: 0.27–1.52), and 0.55 (95% CI: 0.33–0.90), respectively. Compared to those with controlled BP at Visit 3 but without persistent BP control, the adjusted HR for CVD, CHD, stroke, and heart failure for participants with persistent BP control was 0.70 (95% CI: 0.44–1.12), 0.77 (95% CI: 0.36–1.66), 0.64 (95% CI: 0.26–1.59), and 0.53 (95% CI: 0.32–0.90), respectively (**S5 Table in S1 File**).

**Table 4:** Incidence rates and adjusted hazard ratios for cardiovascular events among participants with versus without persistent blood pressure control.

				Hazard ratio (95% confidence interval)	p-value	Hazard ratio (95% confidence interval)	p-value
	Events	Incidence rate*		Model 1		Model 2	
**Cardiovascular disease**							
Non-persistent BP Control	81	25.6 (20.1–31.2)		1 (Ref)		1 (Ref)	
Persistent BP Control	46	13.7 (9.8–17.7)		0.64 (0.44–0.92)	0.015	0.71 (0.46–1.10)	0.125
**Coronary heart disease**							
Non-persistent BP Control	32	9.3 (6.1–12.6)		1 (Ref)		1 (Ref)	
Persistent BP Control	19	5.4 (2.9–7.8)		0.65 (0.36–1.14)	0.134	0.68 (0.34–1.34)	0.263
**Stroke**							
Non-persistent BP Control	25	7.1 (4.3–9.9)		1 (Ref)		1 (Ref)	
Persistent BP Control	11	3.0 (1.2–4.8)		0.52 (0.25–1.05)	0.069	0.65 (0.27–1.52)	0.319
**Heart failure**							
Non-persistent BP Control	64	19.0 (14.3–23.6)		1 (Ref)		1 (Ref)	
Persistent BP Control	33	9.2 (6.1–12.4)		0.57 (0.37–0.87)	0.010	0.55 (0.33–0.90)	0.019

BP = blood pressure.

*Incidence rate per 1,000 person-years (95% confidence intervals).

Model 1 adjusted for age and sex.

Model 2 adjusted for age, sex, diabetes, current smoking, systolic blood pressure, total cholesterol, and high-density lipoprotein.

## Discussion

Less than half of JHS participants taking antihypertensive medication at baseline maintained persistent BP control over a median of 8 years of follow-up. Several participant characteristics were associated with a higher likelihood of persistent BP control including younger age, maintaining family income ≥$25,000 a year, and visiting a health professional in the year prior to each study visit. Persistent BP control was associated with a lower risk for heart failure.

Overall, 48.9% of participants had persistent BP control in the current study. Secondary analyses of BP lowering trials have reported a high proportion of adults do not have persistent BP control [10, 15–17]. A prior study using data from ALLHAT reported that overall, only 20.0% of participants had controlled BP at eight study visits, conducted over a 22 month period [10]. Analyses of data from the Valsartan Antihypertensive Long-term use Evaluation trial, ALLHAT, the International Verapamil SR-Trandolapril Study, and the Coronary Disease Trial Investigating Outcome with Nifedipine report the proportion of participants with controlled BP <140/90 mm Hg at ≥75% of study visits was 33.9%, 36.4%, 36.8%, and 51.5%, respectively [10, 15–17]. Randomized trials often enroll high risk participants who may have BP that is hard to control. However, together with data from the current community-based study, these data highlight the substantial treatment gap in care for adults taking antihypertensive medication. A number of evidence-based approaches are available to increase BP control. The US Surgeon General’s Call to Action to Control Hypertension recommends that clinicians connect patients with community resources to assist in controlling BP, address low rates of antihypertensive medication adherence by utilizing electronic prescribing and 90-day refills, and also counsel patients on how to use home BP monitors and transmit BP readings to a clinical care team [18]. These factors may aid in achieving persistent BP control.

Participants ≥65 years of age were less likely than their younger counterparts to have persistent BP control. BP is more difficult to control among older adults due to factors including arterial stiffening and vascular injury [19–21]. However, prior studies report that a high proportion of older adults can achieve guideline-recommended BP levels, but that BP may be undertreated in this population due to concerns about side effects [6, 19, 22]. Randomized controlled trials have found benefit and little harm from intensive BP treatment among older adults [6, 23, 24]. Among participants ≥75 years in the Systolic Blood Pressure Intervention Trial [23], lowering BP to a target of 120 mm Hg (i.e., intensive) compared with 140 mm Hg (i.e., standard) reduced the risk for CVD by 34% (HR 0.66; 95% CI 0.51–0.85) [23]. There was no difference in number of severe adverse events, including injurious falls, between the intensive and standard treatment groups. Therefore, the benefit of treating BP to guideline recommended levels among older adults outweigh the potential harm.

Having an income ≥$25,000 per year was associated with increased likelihood of persistent BP control. A prior study reported that adults receiving care at ALLHAT study sites in the lowest income quintile (median income $21,800) had a 52% lower odds of BP control at year six of the trial (odds ratio 0.48; 95% CI 0.37–0.63) compared to those in the highest income quintile (median income $49,600) [25]. Policy makers should consider addressing low income as a public health priority given consistent evidence of the association between low income and increased BP [25, 26]. Providing opportunities for people to move from high- to low-poverty neighborhoods may be a potential way to address the effects of low income on BP control as relocation has been associated with a 5 mm Hg decrease in SBP [27, 28]. Healthcare system level interventions may improve BP control for adults with low income [29, 30]. Adapting an evidence-based hypertension treatment protocol used by Kaiser Permanente health system [29] to 12 safety-net clinics resulted in an increase in BP control rates from 60% to 66% among black adults [30]. Also, using existing infrastructure in predominantly black neighborhoods can improve BP control [31]. For instance, a pharmacist-led BP intervention administered to black men in barbershops lowered SBP by 21.6 mm Hg compared to a control group [31].

Attending a visit to a health professional in the year prior to each study visit was associated with a 21% increased likelihood of persistent BP control. The association between visits to a health professional in the past year and rates of BP control have been reported in prior studies [7, 32]. The Affordable Care Act expanded healthcare coverage to millions of adults in the US and resulted in an increase in antihypertensive medication use in states that expanded Medicaid [33, 34]. Additional state and federal health insurance expansions should be considered in the future in order to increase persistent BP control and prevent CVD.

Persistent BP control in the current study was associated with a lower risk for heart failure. A study using ALLHAT data reported an increased risk for CHD, stroke, and heart failure among participants with BP control at <50% compared with 100% of visits [10]. The risk of heart failure in the current study was lower for participants with persistent BP control compared to those with controlled BP at Visit 3 but without persistent BP control. While prior studies have reported a stronger association between cumulative exposure to high BP and CVD risk compared to BP measurements obtained at a single time point [4], the current study demonstrates that assessing long-term BP control may also be more informative for assessing CVD risk than measuring BP control at a single time point.

Strengths of the current analysis include the use of a well-characterized, community-based cohort study with standardized BP measurements at multiple visits, and adjudicated CVD events. Data were available to investigate a large number of factors with persistent BP control. However, the current study has several potential limitations. Generalizability of results may be limited as the JHS only included African Americans from the Jackson, MS metropolitan area. Statistical power to detect an association between persistent BP control and CVD events was limited due to the low number of CVD events. Only six years of CVD event follow-up were available after Visit 3. Additionally, adults with CVD events prior to Visit 3 were excluded from the CVD events analysis, lowering the overall CVD risk of the sample. The number of CVD events in the sample limited our ability to examine the association of BP control at different time points with CVD outcomes (i.e., the association of BP control at Visit 1 only versus at Visit 1 and Visit 2 with CVD outcomes) in comparison to persistent BP control. We were able to utilize a time-varying approach for antihypertensive medication adherence. However, due to the complexity of prescribing patterns over time, we only adjusted for the number of antihypertensive medications at baseline. Finally, while we conducted multivariable adjusted analyses, the study design was observational. Therefore, we cannot rule out the potential of residual confounding.

In conclusion, less than half of the African-American participants in the current study taking antihypertensive medication had persistent BP control. Having persistent BP control has the potential to lower the risk of heart failure events. Efforts to facilitate persistent BP control including ensuring adults with hypertension have a usual source of care, utilizing more intensive antihypertensive therapy among older adults, and improving quality of care among adults with low income through guideline informed standardized care and community outreach should be a public health priority.

## Supporting information

S1 File (DOCX)
