# Supplementary material for: Prevalence, risk factors, and cardiovascular disease outcomes associated with persistent blood pressure control: The Jackson Heart Study
Source: PLoS One. 2022 Aug 5;17(8):e0270675. doi: 10.1371/journal.pone.0270675 (PMC9355196; doi:10.1371/journal.pone.0270675)
Supplement: S1 File — (DOCX) [file pone.0270675.s001.docx]

**(Supplement) Prevalence, risk factors and cardiovascular outcomes associated with persistent blood pressure control: The Jackson Heart Study**

Gabriel S. Tajeu, DrPH,^1^ Calvin L. Colvin, MSPH,^2^ Shakia T. Hardy, PhD,^2^ Adam P. Bress, PharmD, MS,^3^ Bamba Gaye, PhD,^4^ Byron C. Jaeger, PhD,^5^ Gbenga Ogedegbe,^6^ Swati Sakhuja, MPH,^2^ Mario Sims, PhD,^7^ Daichi Shimbo, MD,^8^ Emily C. O’brien, PhD,^9^ Tanya M. Spruill, PhD,^6^ Paul Muntner, PhD^2^

^1^Department of Health Services Administration and Policy, Temple University, Philadelphia, PA

^2^Department of Epidemiology, University of Alabama at Birmingham, Birmingham, AL

^3^Department of Population Health Sciences, University of Utah School of Medicine, Salt Lake City, UT

^4^INSERM, U970, Paris Cardiovascular Research Center, Department of Epidemiology, Paris, France, and Université Paris Descartes, Sorbonne Paris Cité, Faculté de Médecine, Paris, France

^5^Department of Biostatistics, University of Alabama at Birmingham, Birmingham, AL

^6^Department of Population Health, NYU Grossman School of Medicine, New York, NY

^7^Department of Medicine, University of Mississippi Medical Center, Jackson, MS

^8^Department of Medicine, Columbia University Irving Medical Center, New York, NY

^9^Department of Neurology, Duke University School of Medicine, Durham, NC

**Correspondence to:**

Gabriel S. Tajeu

Temple University College of Public Health

Ritter Annex Suite 527
1301 Cecil B. Moore Ave.
Philadelphia, PA 19122-6091

T: 215-204-6457

[gabriel.tajeu@temple.edu](mailto:gabriel.tajeu@temple.edu)

**Supplemental Table 1**. Data collection and definitions of variables included in the current analysis.

| Variable | Visit(s) with variable assessed | | | Definition | Definition of ideal |
| --- | --- | --- | --- | --- | --- |
|  | 1 | 2 | 3 |  |  |
| Age^†^ | ✓ |  | ✓ | Age in years on the date of the study visit. | NA |
| Sex | ✓ |  |  | Women or men. | NA |
| Family income | ✓ |  | ✓ | Self-reported family income less than $25,000 a year or $25,000 a year and higher. | Ideal was defined as a family income ≥$25,000. |
| Education level | ✓ |  |  | Less than high school level education or high school level education and higher.  Less than high school education was defined as <12^th^ grade completed and no GED. | NA |
| Marital status | ✓ |  |  | Married or not married.  Not married was defined by being divorced, separated, widowed or never being married. | NA |
| Number of antihypertensive medical classes taken | ✓ |  |  | Taking 1, 2, 3, or 4 or more different classes of antihypertensive medications.  Antihypertensive medications were recorded during a pill bottle review collected at the study visit. | NA |
| Antihypertensive medication adherence | ✓ | ✓ | ✓ | Adherent or not adherent.  For each medication included on the pill bottle review, participants were asked “Did you take this medication in past 24 hours”? Adherence was defined by self-reported taking of all antihypertensive medications included on the pill bottle review in the prior 24 hours. | Ideal was defined as being adherent. |
| Body mass index | ✓ | ✓ | ✓ | Idea or not ideal.[1]  Calculated as weight (kg)/height (m^2^). Trained staff measured participant weight and height at each exam visit. | Ideal was defined as a body mass index <25 kg/m^2^. |
| Glycemic control | ✓ | ✓ | ✓ | Ideal or not ideal.[1]  Measured after a ≥8 hours fast by the glucose oxidase colorimetric method using a Vitros 950 or 250 (Ortho Clinical Diagnostics analyzer; Ortho Clinical Diagnostics, Raritan, NJ) at Visit 1 using a Roche Modular P Chemistry analyzer (Roche Diagnostics, Indianapolis, IN) at Visits 2 and 3. In addition, fasting Hemoglobin A1c at each Visit was measured using the TOSOH high-performance liquid chromatography system. | Ideal was defined as having a fasting blood glucose <126 mg/dL, a hemoglobin A1c <6.5%, and not taking glucose-lowering medication. |
| Diabetes^†^ |  |  | ✓ | Yes or no.  Participants were categorized as diabetic if they had a fasting blood glucose ≥126 mg/dL or hemoglobin A1c ≥6.5% or were taking glucose-lowering medication. | NA |
| Total cholesterol^†^ |  |  | ✓ | Measured by the cholesterol oxidase method using a Roche Modular P chemistry analyzer (Roche Diagnostics, Indianapolis, IN) | NA |
| High-density lipoprotein cholesterol^†^ |  |  | ✓ | Measured by the magnesium/dextran sulfate precipitation method using a Roche Modular P chemistry analyzer (Roche Diagnostics, Indianapolis, IN). | NA |
| Cigarette smoking^†^ | ✓ |  | ✓ | Non-smoker or current smoker.  Smoking status at Visit 3 determined using self-reported smoking at the nearest preceding annual follow-up call. | Non-smoker |
| Alcohol consumption | ✓ |  | ✓ | No alcohol consumption or any alcohol consumption. | Ideal is defined as no alcohol consumption. |
| Physical activity | ✓ |  | ✓ | Ideal or not ideal[1]  Ascertained using a 30-item modified Baecke questionnaire.[2] Participants were asked the following questions about 3 activities they engaged in the past year:  “Which sport or exercise do you do most frequently?”  “How many months did you do this activity?”  “How many hours a week did you do this activity?”  Responses were used to calculate exercise intensity and duration. | Ideal is defined by having a calculated minutes/week of activity were ≥150 minutes/week of moderate intensity exercise, or ≥75 minutes/week of vigorous intensity, or ≥150 minutes/week of moderate and vigorous intensity exercise. |
| Insurance status | ✓ |  |  | Insured or not insured.  Participants were considered insured if they reported any insurance coverage. | NA |
| Healthcare visit in the past year | ✓ | ✓ | ✓ | Yes or no.  Assessed by the following question: “When was the last time you went to a doctor or other health professional for a routine physical exam or general check-up; that is when you were not sick or pregnant?”  Participants were categorized as yes if they reported a visit within the past year. | Ideal is defined by a response of “yes.” |
| Difficulty accessing healthcare services | ✓ | ✓ | ✓ | No difficulty or some difficulty.  Assessed by the following question: “Overall, how hard has it been for you to get health services you have needed? Would you say it has been very hard, fairly hard, not too hard, or not hard at all?”  Participants were categorized as having no difficulty if they responded “not hard at all.” | Ideal is defined by a response of “no difficulty.” |
| Weekly stress | ✓ |  |  | Low, moderate, and high.  Assessed using the Weekly Stress Inventory.[3]  Categories were created using tertiles with cut-points determined by the distribution of JHS participants with complete data on weekly stress at Visit 1.[3] | NA |
| Depressive symptoms | ✓ |  |  | No and yes.  Assessed using the 20-item Center for Epidemiological Studies Depression Scale (CES-D).[3]  Participants were categorized as yes if they had a CES-D score ≥16.[4] | NA |
| Anger-in | ✓ |  | ✓ | Low, moderate, and high.  Assessed using an 8-item Spielberger Trait Anger scale.[3]  Categories were created using tertiles with cut-points determined by the distribution of JHS participants with complete data on anger-in at each respective visit. | Ideal is defined by being in the lowest tertile. |
| Anger-out | ✓ |  | ✓ | Low, moderate, and high.  Assessed using an 8-item Spielberger Trait Anger scale.[3]  Categories were created using tertiles with cut-points determined by the distribution of JHS participants with complete data on anger-out at each respective visit. | Ideal is defined by being in the lowest tertile. |
| Daily discrimination | ✓ |  | ✓ | Quartiles 1, 2, 3, and 4.  Assessed using a 9-item questionnaire developed by the Jackson Heart Study.[3,5]  Categories were created using quartiles with cut-points determined by the distribution of JHS participants with complete data on daily discrimination at each respective visit. | Ideal is defined by being in the lowest quartile. |
| ^†^Age at Visit 3, diabetes status, total and high-density lipoprotein cholesterol, and cigarette smoking at Visit 3 were used only for the analysis of cardiovascular disease outcomes.  NA=not applicable | | | | | |

**Supplemental Table 2**. Missing data from Jackson Heart Study participants included in the current analysis (N=1,604).

| Characteristics | Participants with missing data  N (%) |  |
| --- | --- | --- |
|  |  |  |
| Demographic |  |  |
| Age, in years, at Visit 1 | 0 (0.0%) |  |
| Gender | 0 (0.0%) |  |
| Income at Visit 1 | 231 (14.4%) |  |
| Income at Visit 3 | 281 (17.5%) |  |
| Education at Visit 1 | 0 (0.0%) |  |
| Marital status at Visit 1 | 4 (0.3%) |  |
| Clinical factors |  |  |
| Systolic blood pressure at Visit 1 | 0 (0.0%) |  |
| Diastolic blood pressure at Visit 1 | 0 (0.0%) |  |
| Number of antihypertensive medication classes at Visit 1 | 0 (0.0%) |  |
| Body mass index at Visit 1 | 1 (0.1%) |  |
| Body mass index at Visit 2 | 42 (2.6%) |  |
| Body mass index at Visit 3 | 47 (2.9%) |  |
| Diabetes at Visit 1 | 66 (4.1%) |  |
| Diabetes at Visit 2 | 502 (31.3%) |  |
| Diabetes at Visit 3 | 45 (2.8) |  |
| Behavioral factors |  |  |
| Adherence to antihypertensive medication at Visit 1 | 0 (0.0%) |  |
| Smoking at Visit 1 | 9 (0.6%) |  |
| Alcohol consumption at Visit 1 | 6 (0.4%) |  |
| Alcohol consumption at Visit 3 | 4 (0.3%) |  |
| Physical activity at Visit 1 | 0 (0.0%) |  |
| Physical activity at Visit 3 | 18 (1.1%) |  |
| Access to health care |  |  |
| Health insurance at Visit 1 | 2 (0.1%) |  |
| Healthcare visit in the past year at Visit 1 | 5 (0.3%) |  |
| Healthcare visit in the past year at Visit 2 | 0 (0.0%) |  |
| Healthcare visit in the past year at Visit 3 | 18 (1.1%) |  |
| Difficulty in obtaining health services at Visit 1 | 5 (0.3%) |  |
| Difficulty in obtaining health services at Visit 2 | 0 (0.0%) |  |
| Difficulty in obtaining health services at Visit 3 | 18 (1.1%) |  |
| Psychosocial factors |  |  |
| Stress at Visit 1 | 701 (43.7%) |  |
| Depression at Visit 1 | 516 (32.2%) |  |
| Anger-in at Visit 1 | 610 (38.0%) |  |
| Anger-in at Visit 3 | 90 (5.6%) |  |
| Anger-out at Visit 1 | 581 (36.2%) |  |
| Anger-out at Visit 3 | 90 (5.6%) |  |
| Daily discrimination at Visit 1 | 23 (1.4%) |  |
| Daily discrimination at Visit 3 | 119 (7.4%) |  |

**Supplemental Table 3**. Proportion of participants with persistent blood pressure control among those taking antihypertensive medication and with blood pressure control at Visit 1 (n=1,226).

| Characteristics | Proportion with persistent BP control among participants with controlled BP at Visit 1 | p-value |
| --- | --- | --- |
| Overall | 64.0 |  |
| Demographic |  |  |
| Age |  |  |
| <65 years | 67.3 | <0.001 |
| ≥65 years | 55.5 |  |
| Sex |  |  |
| Men | 63.5 | 0.794 |
| Women | 64.3 |  |
| Maintained income ≥$25,000 per year* |  |  |
| No | 59.6 | 0.004 |
| Yes | 69.0 |  |
| High school education |  |  |
| No | 55.0 | 0.003 |
| Yes | 65.9 |  |
| Marital status |  |  |
| Married | 65.7 | 0.207 |
| Not married | 62.2 |  |
| Clinical factors |  |  |
| Number of antihypertensive medication classes |  |  |
| 1 | 64.1 | 0.779 |
| 2 | 64.1 |  |
| 3 | 65.8 |  |
| 4+ | 60.0 |  |
| Maintained ideal body mass index* |  |  |
| No | 64.7 | 0.881 |
| Yes | 66.0 |  |
| Ideal glycemic control* |  |  |
| No | 64.3 | >0.999 |
| Yes | 64.4 |  |
| Behavioral factors |  |  |
| Maintained adherence to antihypertensive medication* |  |  |
| No | 62.8 | 0.273 |
| Yes | 66.0 |  |
| Smoking |  |  |
| Non-Smoker | 63.8 | 0.512 |
| Smoker | 67.4 |  |
| Maintained ideal alcohol consumption* |  |  |
| No | 66.7 | 0.073 |
| Yes | 61.7 |  |
| Maintained ideal physical activity* |  |  |
| No | 64.2 | 0.823 |
| Yes | 62.8 |  |
| Access to health care |  |  |
| Health insurance |  |  |
| Uninsured | 60.3 | 0.416 |
| Insured | 64.4 |  |
| Reported visiting a healthcare professional in the past year at each study visit* |  |  |
| No | 58.5 | 0.010 |
| Yes | 66.4 |  |
| Maintained no difficulty in obtaining health services* |  |  |
| No | 62.4 | 0.186 |
| Yes | 66.1 |  |
| Psychosocial factors |  |  |
| Stress |  |  |
| Low | 65.6 | 0.894 |
| Moderate | 63.6 |  |
| High | 65.2 |  |
| Depression |  |  |
| No depressive symptoms | 64.5 | 0.589 |
| Depressive symptoms | 62.1 |  |
| Maintained ideal anger-in* |  |  |
| No | 63.3 | 0.525 |
| Yes | 66.7 |  |
| Maintained ideal anger-out* |  |  |
| No | 63.6 | 0.664 |
| Yes | 66.4 |  |
| Maintained low levels of daily discrimination* |  |  |
| No | 64.4 | 0.237 |
| Yes | 59.3 |  |
| BP: blood pressure  *These factors were available at multiple study visits. For each study visit where these variables were available, we categorized participants as having ideal or non-ideal levels of each of these factors. We then categorized participants as maintaining ideal levels of each factor if participants were in the ideal category at all visits in which they were collected. **Supplemental Table 1** lists these study variable definitions, visits at which they were collected, collection methods, and their classification for ideal level status.  Stress scale tertile cut points: low (0-31), moderate (32-80), high (81-482) | | |

**Supplemental Table 4.** Risk ratios for persistent blood pressure control among participants taking antihypertensive medication and with controlled blood pressure at Visit 1 (n=1,226).

| Characteristic | Model 1  Risk ratios (95% CI) | Model 2  Risk ratios (95% CI) |
| --- | --- | --- |
| Demographic |  |  |
| Age: ≥65 years compared to <65 years | 0.82 (0.74-0.92) | 0.85 (0.76-0.95) |
| Sex: women vs. men | 1.01 (0.92-1.11) | 1.04 (0.94-1.15) |
| Maintained ideal income: yes vs. no | 1.14 (1.04-1.26) | 1.09 (0.98-1.22) |
| High school education: yes vs. no | 1.14 (1.00-1.30) | 1.06 (0.92-1.23) |
| Marital status: married vs. not married | 1.06 (0.97-1.15) | 1.02 (0.93-1.12) |
| Clinical factors |  |  |
| Number of antihypertensive medication classes |  |  |
| 1 | Ref | Ref |
| 2 | 1.00 (0.90-1.10) | 1.01 (0.91-1.12) |
| 3 | 1.03 (0.91-1.16) | 1.04 (0.92-1.17) |
| 4+ | 0.93 (0.79-1.11) | 0.94 (0.79-1.11) |
| Maintained ideal body mass index:  yes vs. no | 1.05 (0.86-1.29) | 1.06 (0.86-1.31) |
| Maintained ideal glycemic control: yes vs. no | 0.96 (0.83-1.11) | 0.94 (0.81-1.08) |
| Behavioral factors |  |  |
| Maintained adherence to antihypertensive medication: yes vs. no* | 1.04 (0.95-1.13) | 1.03 (0.94-1.12) |
| Cigarette smoking: smoker vs. non-smoker | 1.02 (0.88-1.18) | 1.04 (0.89-1.21) |
| Maintained ideal alcohol consumption: yes vs. no | 0.95 (0.87-1.04) | 0.96 (0.88-1.05) |
| Maintained ideal physical activity: yes vs. no | 0.98 (0.83-1.15) | 0.95 (0.81-1.12) |
| Access to health care |  |  |
| Health insurance: insured vs. uninsured | 1.12 (0.96-1.31) | 1.06 (0.90-1.25) |
| Maintained ideal access to primary care: yes vs. no | 1.16 (1.05-1.27) | 1.14 (1.03-1.26) |
| Maintained no difficulty in obtaining health services: yes vs. no | 1.06 (0.97-1.15) | 1.01 (0.92-1.10) |
| Psychosocial factors |  |  |
| Stress |  |  |
| Low | Ref | Ref |
| Moderate | 0.97 (0.85-1.10) | 0.98 (0.86-1.12) |
| High | 1.00 (0.87-1.14) | 1.03 (0.89-1.19) |
| Depression: depressive symptoms vs. no depressive symptoms | 0.95 (0.85-1.08) | 0.99 (0.86-1.14) |
| Maintained ideal anger-in: yes vs. no | 1.07 (0.95-1.21) | 1.05 (0.92-1.19) |
| Maintained ideal anger-out: yes vs. no | 1.06 (0.93-1.22) | 1.07 (0.93-1.22) |
| Maintained ideal levels of daily discrimination: yes vs. no | 0.98 (0.86-1.12) | 0.99 (0.86-1.14) |
| CI: Confidence interval  Model 1 adjusted for age and sex.  Model 2 adjusted for all variables listed in the table.  *These factors were available at multiple study visits. For each study visit where these variables were available, we categorized participants as having ideal or non-ideal levels of each of these factors. We then categorized participants as maintaining ideal levels of each factor if participants were in the ideal category at all visits in which they were collected. **Supplemental Table 1** lists these study variable definitions, visits at which they were collected, collection methods, and their classification for ideal level status.  Stress scale tertile cut points: low (0-31), moderate (32-80), high (81-482) | | |

**Supplemental Table 5**. Incidence rates and hazard ratios and 95% confidence intervals for incident cardiovascular disease events associated with controlled blood pressure at visit 3 but without persistent blood pressure control, uncontrolled blood pressure at visit 3 and persistent blood pressure control.

|  |  |  | Hazard ratio (95% confidence interval) | |
| --- | --- | --- | --- | --- |
|  | N Events | Incidence rate* | Model 1 | Model 2 |
| **Cardiovascular disease** | | | | |
| Controlled BP at Visit 3 without persistent BP control | 31 | 24.3 (15.7 – 32.8) | 1 (Ref) | 1 (Ref) |
| Persistent BP Control | 46 | 13.7 (9.8 – 17.7) | 0.66 (0.42 – 1.04) | 0.70 (0.44 – 1.12) |
| **Coronary heart disease** | | | | |
| Controlled BP at Visit 3 without persistent BP control | 11 | 7.8 (3.2 – 12.5) | 1 (Ref) | 1 (Ref) |
| Persistent BP Control | 19 | 5.4 (2.9 – 7.8) | 0.76 (0.36 – 1.60) | 0.77 (0.36 – 1.66) |
| **Stroke** | | | | |
| Controlled BP at Visit 3 without persistent BP control | 9 | 6.3 (2.2 – 10.5) | 1 (Ref) | 1 (Ref) |
| Persistent BP Control | 11 | 3.0 (1.2 – 4.8) | 0.57 (0.24 – 1.39) | 0.64 (0.26 – 1.59) |
| **Heart failure** | | | | |
| Controlled BP at Visit 3 without persistent BP control | 29 | 21.0 (13.4 – 28.7) | 1 (Ref) | 1 (Ref) |
| Persistent BP Control | 33 | 9.2 (6.1 – 12.4) | 0.51 (0.31 – 0.85) | 0.53 (0.32 – 0.90) |
| BP: blood pressure.  *Incidence rate per 1,000 person-years (95% confidence intervals).  Model 1 adjusts for age and sex.  Model 2 adjusts for age, sex, diabetes, current smoking, systolic blood pressure, total cholesterol, and high-density lipoprotein cholesterol. | | | | |

**Supplemental Figure.** Consort diagram.

**Exclusions**

2,854 participants were not taking antihypertensive medication at Visit 1 or had missing Visit 1 SBP or DBP.

491 participants did not attend Visit 2.

10 participants had missing Visit 2 SBP or DBP.

343 participants did not attend Visit 3.

4 participants had missing Visit 3 SBP or DBP.

213 participants had a history of CVD at Visit 1.

135 participants did not consent to event follow-up.

JHS participants at Visit 1

N = 5,306

JHS participants taking antihypertensive medication* and with complete information on SBP and DBP at Visit 1

N = 2,452

JHS participants who attended Visit 2

N = 1,961

JHS participants with complete information on SBP and DBP at Visit 2

N = 1,951

JHS participants who attended Visit 3

N = 1,608

JHS with complete information on SBP and DBP at Visit 3

N = 1,604

JHS participants consenting to CVD event follow-up at Visits 1, 2, and 3

N = 1,256

No history of CHD during follow-up

N= 1,203

No history of stroke during follow-up

N= 1,229

No history of heart failure during follow-up

N= 1,212

JHS: Jackson Heart Study; SBP: Systolic blood pressure; DBP: Diastolic blood pressure; CVD: Cardiovascular disease; CHD: coronary heart disease.

*Not taking antihypertensive medication was defined as self-reporting not taking antihypertensive medication or having a missing response on the question assessing self-reported antihypertensive medication use, or not having an antihypertensive medication identified on the pill bottle review.

No history of any CVD during follow-up

N= 1,151

JHS without a history of CVD at Visit 1

N = 1,391

**Supplemental Sources Cited**

1. Lloyd-Jones DM, Hong Y, Labarthe D, Mozaffarian D, Appel LJ, Van Horn L, Greenlund K, Daniels S, Nichol G, Tomaselli GF, Arnett DK, Fonarow GC, Ho PM, Lauer MS, Masoudi FA, Robertson RM, Roger V, Schwamm LH, Sorlie P, Yancy CW and Rosamond WD. Defining and setting national goals for cardiovascular health promotion and disease reduction: the American Heart Association's strategic Impact Goal through 2020 and beyond. *Circulation*. 2010;121:586-613.

2. Dubbert PM, Carithers T, Ainsworth BE, Taylor Jr HA, Wilson G and Wyatt SB. Physical activity assessment methods in the Jackson Heart Study. *Ethn Dis*. 2005;15:S6-56.

3. Payne TJ, Wyatt SB, Mosley TH, Dubbert PM, Guiterrez-Mohammed ML, Calvin RL, Taylor HA, Jr. and Williams DR. Sociocultural methods in the Jackson Heart Study: conceptual and descriptive overview. *Ethn Dis*. 2005;15:S6-38-48.

4. WEISSMAN MM, SHOLOMSKAS D, POTTENGER M, PRUSOFF BA and LOCKE BZ. ASSESSING DEPRESSIVE SYMPTOMS IN FIVE PSYCHIATRIC POPULATIONS: A VALIDATION STUDY. *American Journal of Epidemiology*. 1977;106:203-214.

5. Sims M, Wyatt SB, Gutierrez ML, Taylor HA and Williams DR. Development and psychometric testing of a multidimensional instrument of perceived discrimination among African Americans in the Jackson Heart Study. *Ethn Dis*. 2009;19:56-64.
